# Analysis of melatonin regulation of germination and antioxidant metabolism in different wheat cultivars under polyethylene glycol stress

**DOI:** 10.1371/journal.pone.0237536

**Published:** 2020-08-13

**Authors:** Dongxiao Li, William D. Batchelor, Di Zhang, Hanxiao Miao, Hongye Li, Shijia Song, Ruiqi Li

**Affiliations:** 1 College of Agronomy, State Key Laboratory of North China Crop Improvement and Regulation, Key Laboratory of Crop Growth Regulation of Hebei Province, Hebei Agricultural University, Baoding, Hebei Province, China; 2 Biosystems Engineering Department, Auburn University, Auburn, Alabama, United States of America; 3 Hebei Academy of Agriculture and Forestry Sciences, Shijiazhuang, China; University of Melbourne, AUSTRALIA

## Abstract

Melatonin is effective in enhancing various abiotic stress resistances of plants. However, its underlying mechanisms in drought-resistance in winter wheat (*Triticum aestivum* L.) is not clear. The goal of this work was to investigate the effect of melatonin on seed germination and to evaluate leaf antioxidant physiology for two wheat varieties. Experiments included 20% PEG, melatonin plus 20% PEG and a control using two contrasting wheat varieties (JM22– drought sensitive and HG35– drought resistant). Melatonin levels were 0, 1, 10, 100 and 300 μmol L^-1^. Results revealed that 300 μmol L^-1^ of melatonin alleviated the negative effect of water stress on germination and increased radicle length, radicle number, and plumule length of the germinated seeds. Principal component analysis showed a significant change in amino acid content during germination and this change was dependent on melatonin concentration and the variety. Lysine (Lys) content in wheat seeds under the PEG plus 300 μmol L^-1^ melatonin treatment increased compared with that of the seeds under PEG alone. There was a significant and positive correlation between Lys content and morphological index of germination. During seedling growth, soluble protein was involved in osmotic adjustment and superoxide dismutase (SOD) activity was increased to mitigate the damage in the cytomembrane of JM 22 leaf under 300 μmol L^-1^ melatonin plus PEG treatment. The effect of melatonin was dependent on SOD activity increasing significantly for HG35—a drought resistant variety. The results of this work lays a foundation for further studies to determine if melatonin can be economically used to mitigate the impact of dry planting conditions on wheat productivity in North China Plain.

## Introduction

Drought is a major abiotic stress that leads to significant yield loss worldwide, and has affected global food security [1, 2]. Wheat (*Triticum durum* L.), is the most important food crop grown in North China Plain (NCP). Rainfall is not adequate for wheat production in the NCP, thus the crop is often irrigated [3]. Yields are under pressure from frequent drought and limited groundwater sources which has limited irrigation [4]. Drought stresses often leads to the accumulation of reactive oxygen species that attack proteins, lipids, carbohydrates and nucleic acids of plants, breaks the physiological balance and leads to reduced photosynthesis and final yield [5]. Strategies to mitigate the impact of drought and limited water on wheat yield would enhance the sustainability of wheat production in the NCP.

Melatonin (*N*-acetyl-5-methoxytryptamine), a derivative of the essential amino acid tryptophan, can trigger plant defense responses against adverse environment stress [6–8]. Studies have reported diverse roles of melatonin in plants, as an abiotic anti-stressor, biotic anti-stressor, biological rhythm regulator, and plant hormone [9–12]. Melatonin applied to cucumber seeds improved their germination rate during chilling stress and water stress [8, 13]. Melatonin was also found to reduce copper toxicity in red cabbage seedlings (*Brassica oleracea* rubrum) [14]. Amino acid metabolism is related to and provides nutrients during seed germination [15, 16]. When adverse stress occurs, the compatible solutes accumulate and help to maintain the turgor pressure and protect the macromolecular structures against water loss [17]. Proteins are easily damaged by reactive oxygen species which cause amino acids such as histidine, tyrosine, methionine, cysteine, and tryptophan to respond to free radicles [18–22]. Melatonin is structurally related to tryptophan and tryptamine, and they share common precursors in the biosynthetic route of tryptophan and tryptamine [23]. Pretreatment of *Medicago sativa* with melatonin provided osmotic protection by regulating proline homeostasis and improved plant tolerance to drought [24]. Zhang et al. [25] reported that melatonin alleviated the inhibitory effects of salinity on the gemination of cucumber seeds, which was possibly related to changes in amino acid content by regulating storage protein degradation. Melatonin also accelerated the metabolic flow from the precursor amino acids, arginine and methionine, to polyamines, which mitigated the effects of salt stress on wheat seedlings [26]. There have been few reports on how amino acid affects wheat seeds during germination under drought stress plus melatonin.

Additional studies have shown different effects of melatonin at different concentrations on seeds germination and seedling growth of wheat [27]. Melatonin at 1 μmol L^-1^ or 10 μmol L^-1^ concentration eliminated the inhibitory effect of copper on the fresh weight of red cabbage seedlings; however, melatonin at 100 μmol L^-1^ concentration had a negative effect on seed germination and seedling growth and enhanced the toxic effect of copper [14]. Priming seed with melatonin at 0.8 mmol L^-1^ significantly improved germination energy, germination percentage, proline content, and total phenolic content of maize [28]. However, it is still not clear how different concentrations of melatonin affect amino acids during seed germination and the effect of melatonin concentration on antioxidant defense responses of different wheat varieties seedling under water stress.

The objectives of this work were (1) to investigate the germination characteristics of two wheat varieties with melatonin at 0, 1, 10, 100, and 300 μmol L^-1^ concentrations under drought stress; (2) evaluate the changes in amino acid content during germination of wheat seeds under different melatonin levels; and (3) to determine the effective concentration of melatonin in increasing antioxidant activity in wheat. This study will provide information about the mechanism of drought resistance in germination and seedling growth of different wheat varieties treated by melatonin.

## Materials and methods

### Tested materials and reagents

Experiments were carried out in the key laboratory of crop growth regulation, Agricultural University of Hebei, Baoding city, China in 2017. Two wheat (*Triticum aestivum* L.) varieties, including the drought-tolerant cultivar ‘Hengguan35’ (HG35) and the irrigated cultivar ‘Jimai22’ (JM22), were used in this study. Seeds of HG35 were provided by the Dry Land Farming Research Institute of Hebei Academy of Agricultural and Forestry Sciences and JM22 seeds were donated by Crop Research Institute, Shandong Academy of Agricultural Sciences. Melatonin and polyethylene glycol 6000 (PEG6000) were purchased from Beijing Sinopharm Chemical Reagent Co., Ltd.

### Preparation of melatonin solution

20% PEG solution (W/V): 20 g polyethylene glycol powder was weighed and dissolved into distilled water and calibrated to 100ml. Requisite amount of melatonin was calculated according to molecular weight and concentrations. Because melatonin is not very soluble in water, it was first diluted by 1mL of ethyl alcohol (95%) and was then added to the 20% PEG solution (is -5.357 bar, calculated with the equation of Michel and Kaufmann [29]) to prepare 0, 1, 10, 100, and 300 μmol L^-1^ of melatonin solutions for treatment.

### Experimental treatments

**Fig 1:**
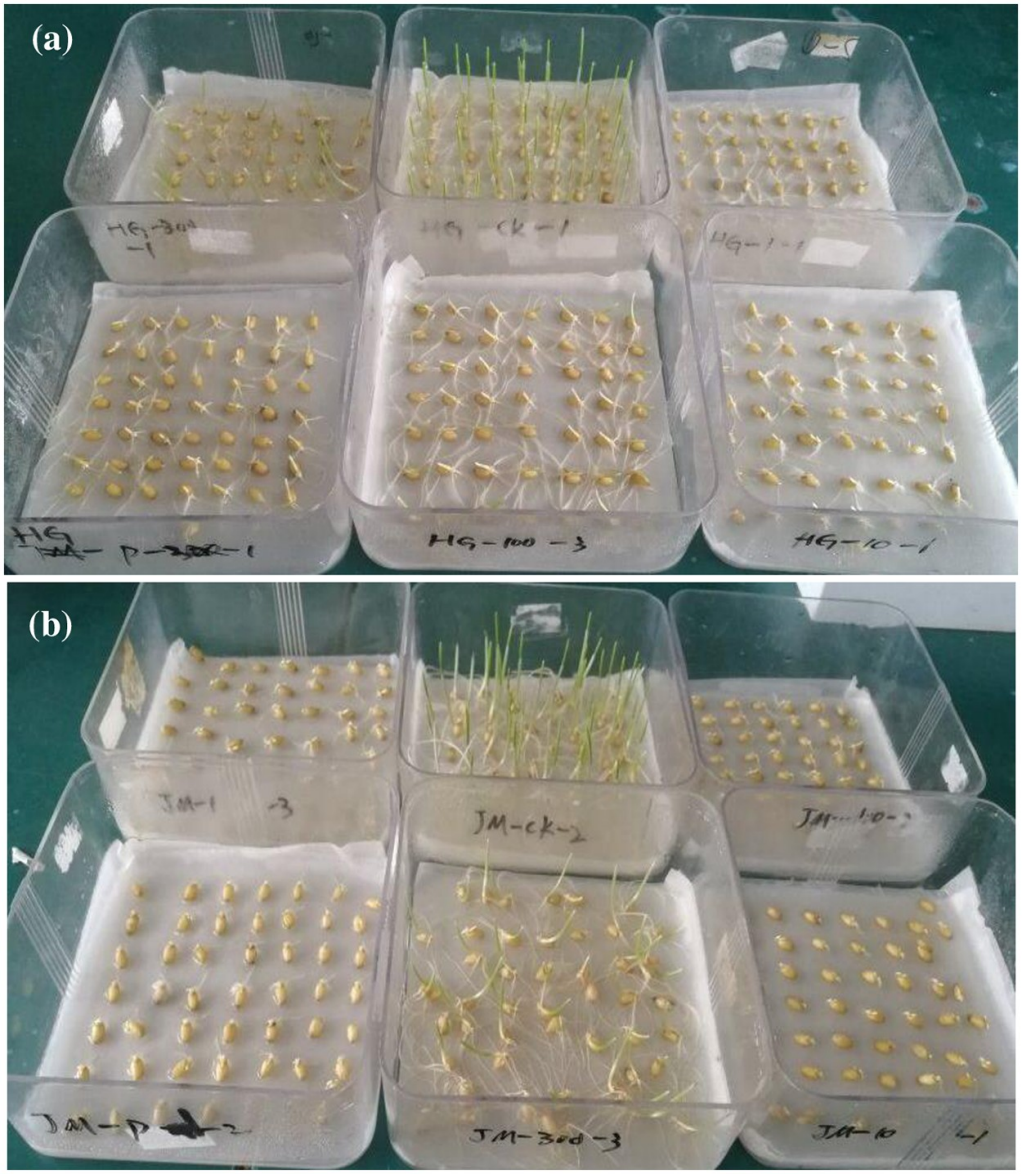
Germination of HG35 (a) and JM22 (b) seeds under different treatments.

Wheat seeds were selected and surface-sterilized with 70% ethanol for two minutes. Seeds were washed several times with distilled water and were placed in a germination box with two layers of filter paper kept saturated with six different treatments as follows: (1) distilled water (control, CK), (2) 20% PEG-6000, (3) 20% PEG plus 1 μmol L^-1^ melatonin (1 μM+20% PEG), (4) 20% PEG plus 10 μmol L^-1^ melatonin (10 μM+20% PEG), (5) 20% PEG plus 100 μmol L^-1^ melatonin (100 μM+20% PEG), and (6) 20% PEG plus 300μmol L^-1^ melatonin (300 μM+20% PEG). Three replicates were maintained for each treatment. Fifty seeds were placed in each germination box and incubated in a growth chamber at day/night temperatures of 20 °C/15 °C in a light/dark regime. Relative humility was 60%. The number of seed germinated was recorded daily up to seven days. Water and other solutions were added into the germination boxes in time to prevent drying (Fig 1).

**Fig 2:**
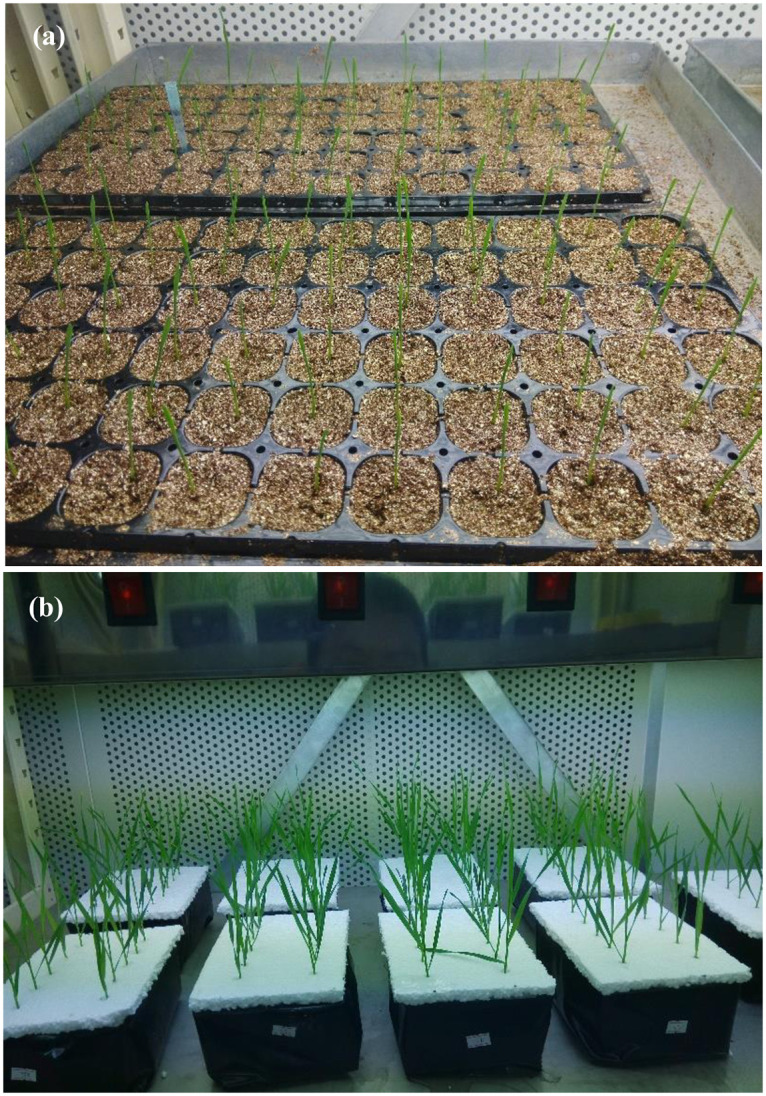
Seed germination (a) and seedling cultivation (B) of two wheat cultivars in a growth chamber.

A second experiment was performed in a growth chamber with the same conditions as described for the germination box experiment. Three treatments including CK, 20%PEG, 20%PEG plus 300 μmol L^-1^ melatonin solution which take obvious improving effect on seed germination were set at three leaf age in liquid MS medium. The selected seeds were germinated in a hole tray filled with saturated-water vermiculite (Fig 2a). When the third leaf appeared, seedlings were transferred into plastic boxes sealed with black film and filled with three treatment solutions (Fig 2b). After seedlings treated for 24 hours, the five replicate leaf samples were collected from each of the three treatments, then quick-frozen using liquid nitrogen, and stored in a -80°C freezer until measurements of antioxidant enzyme activities, soluble protein content, and malondialdehyde content could be made.

### Measurements

#### Morphological index of germination

Germination percentage and germination potential were calculated after seven and three days of incubation according to the following equation:

$$\text{Germination}\;\text{percentage}=\text{the}\;\text{number}\;\text{of}\;\text{germinated}\;\text{seeds}\;\text{on}\;\text{the}\;7^{\text{th}}\;\text{day}/\text{total}\;\text{number}\;\text{of}\;\text{seeds}\;\text{sown}\times 100$$ (1)

$$\text{Germination}\;\text{potential}=\text{the}\;\text{number}\;\text{of}\;\text{germinated}\;\text{seeds}\;\text{on}\;\text{the}\;3^{\text{rd}}\;\text{day}/\text{total}\;\text{number}\;\text{of}\;\text{seeds}\;\text{sown}\times 100$$ (2)

$$\text{Germination}\;\text{index}=\sum \frac{\text{Gt}}{\text{Dt}}$$ (3)

where Gt is the number of germinated seeds after t days, Dt represents germination days.

Plumule length and radical length of germinated seed were measured with a ruler after 72 hours of incubation. Each measurement was repeated five times.

#### Leaf physiological index

Leaf samples of wheat seedling from the growth chamber experiment that were frozen and stored at -80℃ were cut into 0.5 g pieces and were ground in a pre-cooled mortar. The homogenate was diluted with phosphatic buffer solution (50 mM, PH7.8) to a 5 mL mixture, which was centrifuged for 20 mins in a refrigerated centrifuge with a speed of 1000 r/min. The liquid supernatant was put into a 10 mL centrifuge tube to measure the activities of superoxide dismutase (SOD) with nitro blue tetrazolium, peroxidase (POD) with guaiacol colorimetric determination, and catalase (CAT) with ultraviolet spectrophotometer [30]. The content of soluble protein and malondialdehyde (MDA) were measured with coomassie brilliant blue and thiobarbituric acid colorimetric determination [31].

#### Measurement of amino acid content

Seeds germinated after incubation were crushed into a fine powder and sifted through a 100-mesh sieve. A subsample (1.00 g) was hydrolyzed for 14 h at 110 °C in the presence of 10 mL of 6 mol L^-1^ hydrochloric acid. This was further diluted with water to 10 mL at room temperature. About 1 mL of the extraction was transferred into a centrifuge tube and concentrated by drying in a vacuum chamber. The concentrated sample was dissolved in 2 mL of hydrochloric acid (0.1 mol L^-1^) and was detected by a reversed-phase high performance liquid chromatograph (RP-HPLC). The HPLC system (Agilent 1200, USA) consisted of a C18 column with 5 μm particle size (4.6 mm inner diameter, 250 mm length; Komati Universal) and a diode array detector. The mobile phase A was prepared by adding acetonitrile. The mobile phase B was prepared by adding acetic acid-sodium acetate buffer (pH = 5.25 ± 0.05, adjusted using glacial acetic acid) containing 0.03 mol L^-1^ sodium acetate solution and 0.15% triethylamine. The column temperature was set at 40 °C. The detection wavelength used was 360 nm. The flow rate was maintained constant at 1 mL min^-1^, and the column was set at ambient temperature. As many as 42 samples were injected into the column by a G1329A auto injector with a 10 μL sample loop. The gradient elution procedures are shown in Table 1.

**Table 1:** Gradient elution program.

Time/min		0	10	12	25	30	37	39	45
Mobile phase	A	18	18	29	34	55	60	18	18
	B	82	82	71	66	45	40	82	82

#### Aminoacyl derivatization

The standard solution or sample solution (100 μL) was transferred into a centrifuge tube (1.5 mL) and mixed with a buffered solution (200 μL; pH 9.0) and 2,4-dinitrochlorobenzene (100 μL; 300 mg mL^-1^; acetonitrile as solvent). The mixture was vortex-mixed for 1 min and extracted by heating in a 90 °C thermostatic water bath for 90 min in dark. After the reaction, the resulting solution was mixed with 10% (v/v) acetic acid (50 μL, pH adjusted to a neutral value) and diluted with distilled water to 1 mL. The solution was further filtered through an organic membrane.

#### Standard curve development

Standard solutions (500 mg L^-1^) of 17 different amino acids, including aspartic acid (Asp), glutamic (Glu), histidine (His), serine (Ser), arginine (Arg), glycine (Gly), threonine (Thr), proline (Pro), alanine (Ala), valine (Val), methionine (Met), cysteine (Cys), isoleucine (Ile), leucine (leu), phenylalanine (Phe), lysine (Lys), and tyrosine (Tyr), were prepared by diluting the stock solution (amino acid standard mixture,10 mL, 0.1mol L^-1^ HCl) to 5, 50, 125, 100, 150, 200, 300, 400, and 500 mg L^-1^ with 0.1 mol L^-1^ HCl. Prepared standard solutions (100 μL) were used to measure after aminoacyl derivatization performed as discussed above.

### Statistical analysis

Data were analyzed by analysis of variance (ANOVA) with three replicates using Microsoft Excel 2010 and SPSS 19.0 (SPSS Inc., Chicago, USA). The Duncan’s new multiple range (DMR) test at 5% probability level was used to test the differences among the mean values.

## Results

### Seed germination

Germination percentage and germination index for JM22 decreased significantly (12.5% and 48.26%) under PEG treatments compared with the control (Table 2). However, there was no improvement in germination percentage and germination index of JM22 drought stressed seeds with melatonin at any of the tested concentrations (1, 10, or 100 μM). Even the germination percentage and germination index of JM22 seeds with PEG plus melatonin at 10 μM concentration were less significantly than PEG alone. Germination percentage improved significantly with the PEG plus 300 μM melatonin treatment comparing with PEG alone. There was no significant difference in germination percentage between control and PEG plus 300 μM melatonin treatment, whereas the germination index was significantly less under PEG plus 300 μM melatonin treatment compared with the control. Additionally, germination potential was significantly less with 1 μM and 10 μM melatonin treatments, whereas there was no obvious difference among other treatments.

**Table 2:** The effect of different treatments on the germination of wheat seeds.

Treatments		JM 22			HG35	
	Germination Percentage%	Germination index	Germination potential%	Germination Percentage%	Germination index	Germination potential%
Control	96.0a	120.6a	96.0a	98.7a	125.2a	98.7a
PEG	84.0b	62.4c	80.0ab	95.3abc	83.2d	94.7ab
1μM+PEG	78.0b	72.3c	76.7b	88.7cd	97.1c	88.7bc
10μM+PEG	66.7c	37.7d	43.3c	82.7d	81.3d	80.7bc
100μM+PEG	77.3b	76.4c	77.3ab	90.0bcd	108.0bc	89.3b
300μM+PEG	99.3a	100.1b	94.0ab	96.7ab	111.9b	88.7bc

Different letters denote significant differences (p<0.05) within the same factor or interaction on each measurement day. Values are mean ± SE (n = 4). The same lower case letters indicate no significant difference (P < 0.05)

For HG35, germination percentage and germination potential of seeds under PEG treatment did not change significantly compared with the control; however, the germination index was less (33.55%; P<0.05). Germination percentage of seeds with PEG plus 10 μM treatment was the lowest. Germination indexes of seeds with PEG plus 100 μM melatonin and 300 μM melatonin treatments were higher (29.81% and 34.50%, respectively; *p*<0.05) compared with PEG alone.

### Morphological characters of germination

**Fig 3:**
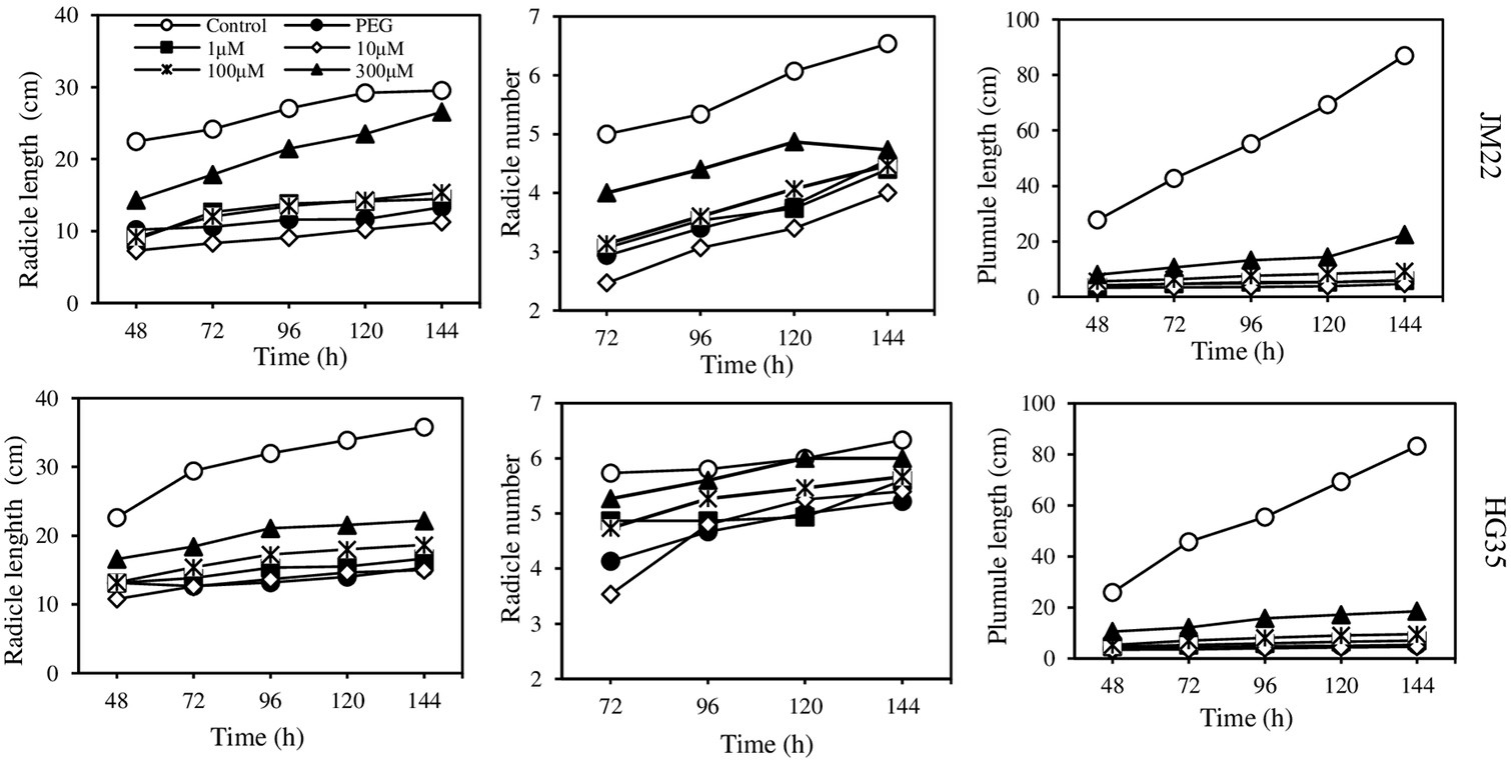
Effects of different concentrations of melatonin on radicle length, radicle number, and plumule length of JM22 and HG35. Values are means of three biological replications.

Radicle length and radicle number of both JM22 and HG35 increased gradually from 72 hours to 144 hours after germination (Fig 3). For JM22, radicle length under PEG alone and PEG plus 10 μM melatonin treatments decreased by 56.57% and 65.25% on average compared with the control. No obvious change was observed under PEG plus 1 μM melatonin and 100 μM melatonin treatments. However, PEG plus 300 μM melatonin treatment resulted in an increase in radicle length (79.01%) compared with PEG alone. Radicle number under other treatments was significantly less than the control, and the least radicle number was recorded under PEG plus 10 μM melatonin treatments (*p*<0.05). Radicle number increased (24.41%) under PEG plus 300 μM melatonin treatment compared with PEG alone. Plumule length decreased significantly under PEG and PEG plus melatonin treatments compared with the control. There was no effect of melatonin on plumule length.

For HG35, radicle length and plumule length under all treatments were significantly less than that of the control. Radicle length under PEG plus 300 μM melatonin treatment was less (34.57%) than that of the control; however, it was more (46.04%) than that of PEG alone. There was no significant difference in radicle number between control and PEG plus 300 μM treatments. The changing in plumule length of HG35 was similar to that of JM22. Additionally, radicle length and radicle number of the drought-resistant variety HG35 were more than that of the irrigated cultivar JM22 under PEG treatment.

### Amino acid content

**Fig 4:**
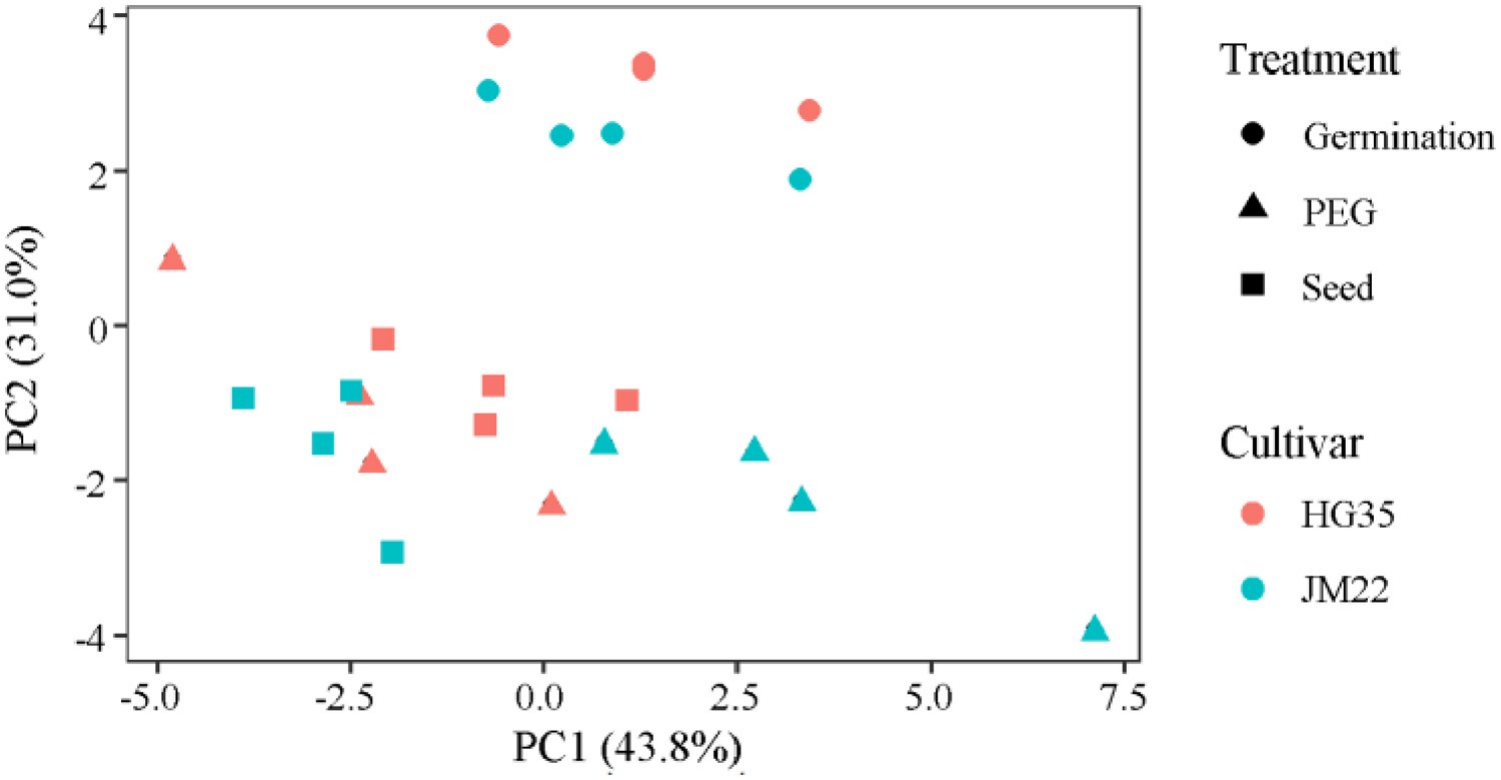
Principal component analysis of amino acid for different wheat cultivars. Germination represent dry seeds germinated by distilled water; PEG represent dry seeds germinated by 20% PEG; Seed represent dry seeds.

Fig 4 showed the variations in amino acid content among all different treatments. Principal component analysis extracted two major components that together accounted for 74.8% of the variance in the data set. Principal component 1 (PC1, X-axis) explained 43.8% of variation among the individual samples, and principal component 2 (PC2, Y-axis) explained 31.0% of variation. After seed germinated, the amino acid content of both cultivars shifted greatly along PC2 axis. After seed germinated with PEG- 6000, the amino acid content of both cultivars shifted down along PC2 axis. Moreover, HG35 and JM22 were separated with each other.

**Fig 5:**
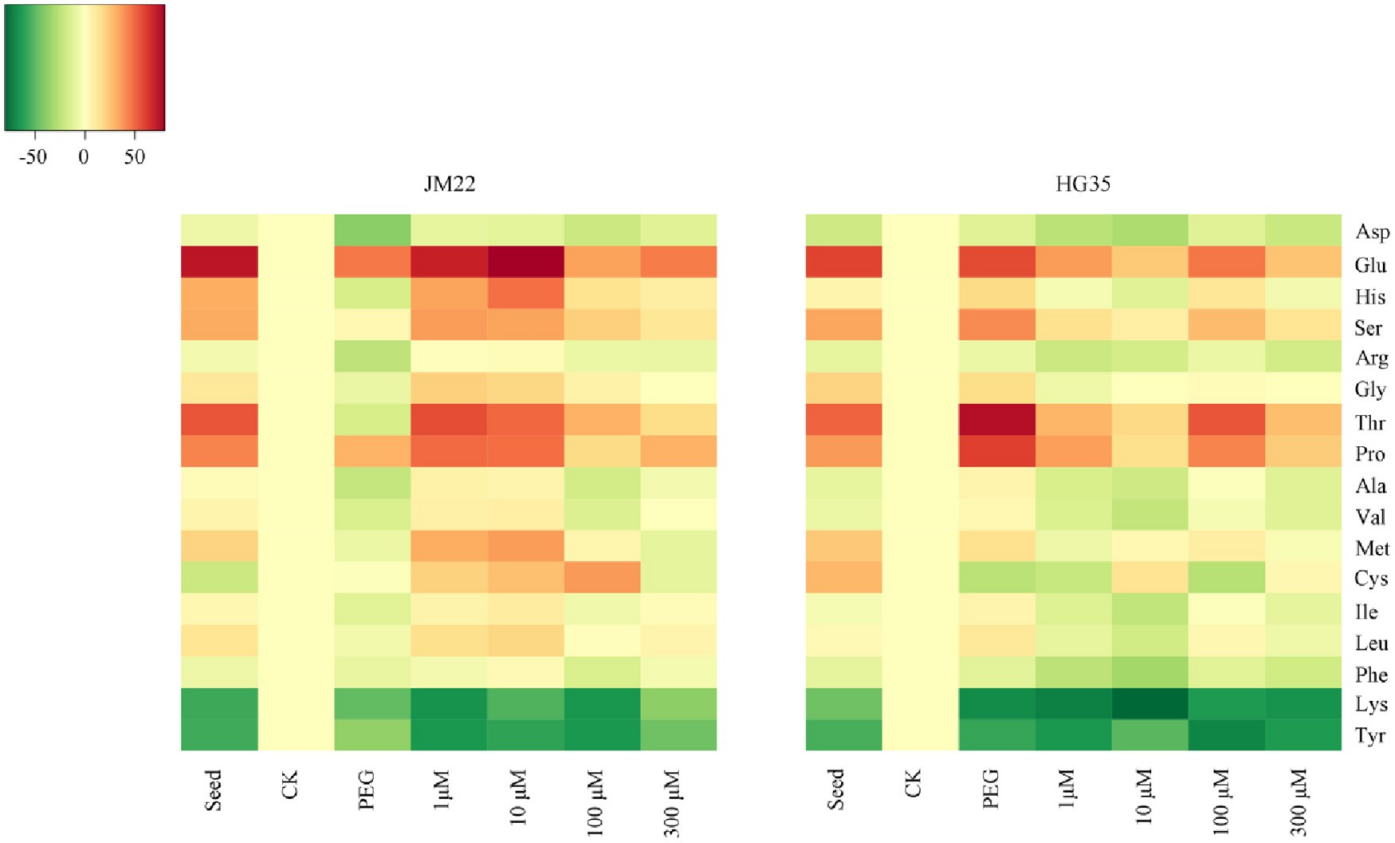
The cluster heatmap of amino acid content differences of two wheat varieties during germination under different treatments. The graph’s horizontal axis shows different treatments (seed represent no-germinated seeds; CK represent seeds germinated by distilled water; PEG represent seeds germinated by 20%PEG; 1μM, 10μM, 100μM, and 300μM, represent seeds germinated by 1μM, 10μM, 100μM, and 300μM melatonin plus 20%PEG, respectively), and the vertical axis shows different amino acid. Color gradients represent the differences value of amino acid contents under other treatments compared with that of CK.

Glu was the highest amino acid detected in both the cultivars followed by Pro. After germination, Glu and Pro contents reduced by 17.16 mg kg^-1^ and 4.41 mg kg^-1^ for JM22 and by 12.96 mg kg^-1^ and 3.56 mg kg^-1^ for HG35, respectively (S1 Table). After germination, Lys and Tyr contents in CK seeds increased by 129.22% and 130.77%, respectively, for JM22, and by 71.85% and 98.59%, respectively, for HG35. Lys and Tyr contents in seeds under other treatments decreased to different levels compared with CK; Glu and Pro contents reversely increased (Fig 5). Amino acid content varied based on melatonin concentration and the cultivar. For JM22, Lys and Tyr contents reduced by 48.44% and 36.67%, respectively, in seeds under PEG compared with CK; however, melatonin plus PEG treatments raised the reduction except 300 μM melatonin reducing Lys content by 37.96%. Glu and Pro contents increased by 44.41% and 29.52% in seeds under PEG treatment compared with CK, and increased by 74.40% and 48.19% under PEG plus melatonin at low concentration (1 μM and 10 μM). For HG35, Lys and Tyr contents reduced by 62.72% and 53.19% under PEG treatment compared with CK; Lys content reduced by 57.97% and 60.13% under PEG plus melatonin at 100 μM and 300 μM treatments, respectively. Glu and Pro contents increased by 51.97% and 55.27% in seeds under PEG alone. The increase in the two indexes was less under PEG plus melatonin treatments at different concentrations.

Compared with PEG alone, the contents of Glu, Met, Cys, and Tyr in JM22 seeds were less under PEG plus melatonin at 300 μM concentration treatment; however, the contents of other amino acids increased (Fig 5 and S1 Table). Cys and Lys contents in HG35 seeds under PEG plus 300 μM melatonin treatment were more than that of PEG alone, whereas other components were lower.

### Correlation analysis

Correlation analysis revealed a positive and significant correlation between Asp content and morphological indexes such as germination index, radical length, and radical number (Table 3). Glu content was negatively correlated with the morphological indexes of germination especially plumule length. After seed germination, Glu and Pro contents reduced significantly (S1 Table and Fig 5). Phe content was significantly and positively correlated with radical number. Lys content was significantly and positively correlated with germination percentage, germination index, germination potential, radicle length, and plumule length.

**Table 3:** The correlation analysis of variance between morphological index of germination and 17 amino acids.

Amino acid	Germination percentage	Germination index	Germination potential	Radicle length	Plumule length	Radicle number
Asp	0.333	0.602*	0.418	0.588*	0.501	0.732**
Glu	-0.364	-0.434	-0.357	-0.497	-0.643*	-0.224
His	-0.175	0.028	-0.14	-0.021	-0.105	0.259
Ser	-0.259	-0.231	-0.263	-0.294	-0.427	-0.042
Arg	-0.028	0.294	0.172	0.28	0.168	0.51
Gly	0.011	0.172	0.067	0.102	-0.042	0.406
Thr	-0.287	-0.196	-0.368	-0.21	-0.252	-0.035
Pro	-0.427	-0.538	-0.525	-0.531	-0.531	-0.371
Ala	0.049	0.161	0.077	0.182	0.105	0.35
Val	0.126	0.217	0.147	0.203	0.098	0.434
Met	-0.459	-0.305	-0.267	-0.287	-0.371	-0.091
Cys	0.084	0.231	0.252	0.091	-0.189	0.308
Ile	0.042	0.161	0.063	0.126	0.049	0.42
Leu	-0.042	0.098	-0.004	0.077	-0.042	0.336
Phe	0.280	0.413	0.414	0.410	0.305	0.585*
Lys	0.599*	0.609*	0.584*	0.673*	0.680*	0.522
Tyr	0.278	0.176	0.340	0.162	0.246	0.165

* indicate significant correlation (P > 0.05).

### Soluble protein and malondialdehyde contents

**Fig 6:**
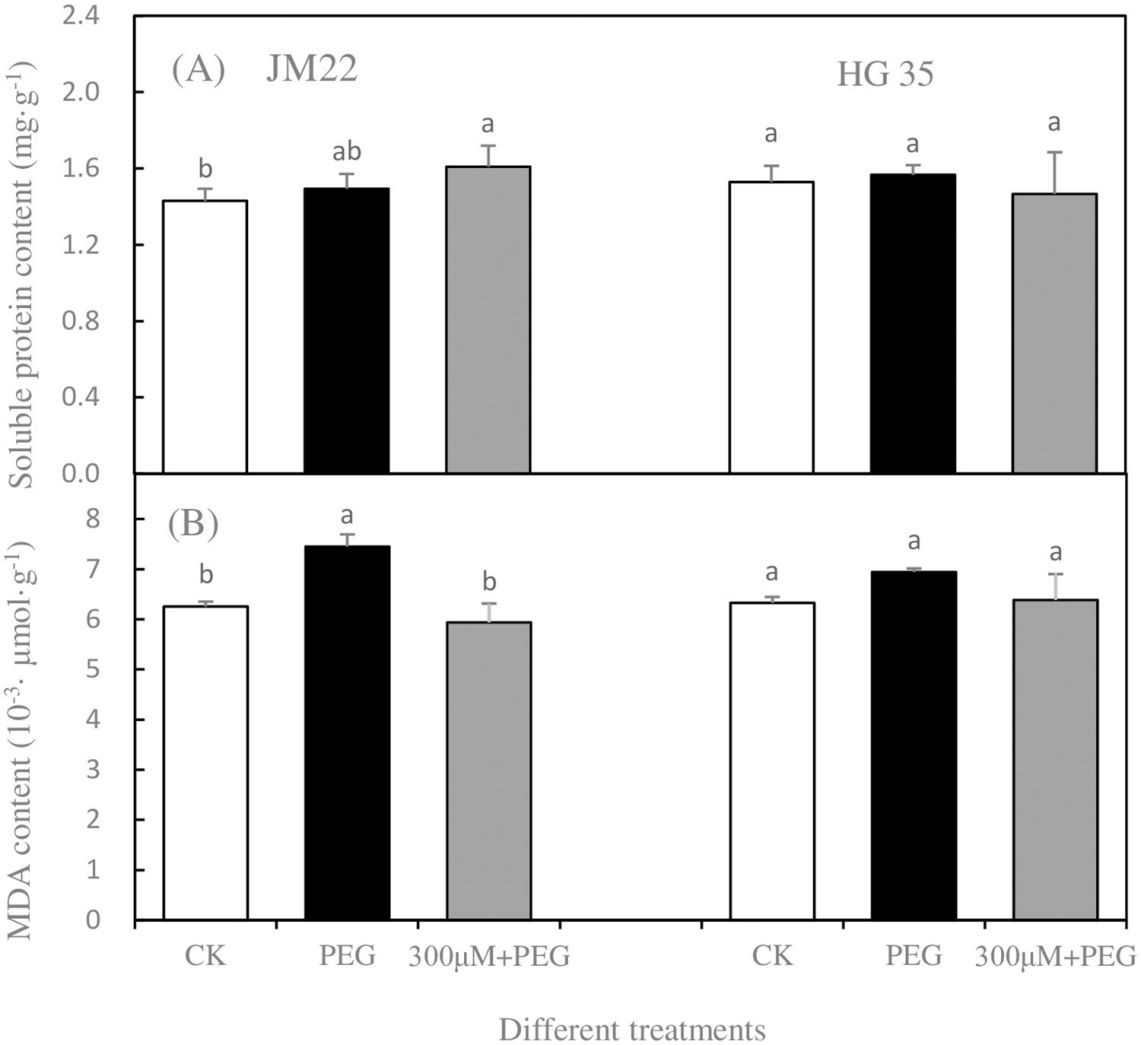
The effect of 300 μM melatonin on soluble protein content (A) and MDA content(B) of wheat seedling leaf under different treatments. Different letters denote significant differences (p<0.05) within the same factor or interaction. Bars indicate mean relative expression values ± SE (n = 4).

Fig 6A showed changes in soluble protein content in leaf tissue of two varieties of wheat seedlings. For JM22, soluble protein content increased insignificantly in the PEG treatment compared with CK; however, this value increased significantly by 12.57% in the PEG+300 μM treatment. For HG35, there was no obvious differences in soluble protein content in leaf tissue among all treatments.

Fig 6B showed that MDA content in leaf of JM 22 was 19.12% higher in PEG treatment compared to CK. Under PEG plus 300 μM melatonin, MDA content decreased to the same level as CK. For HG35, no obvious change in MDA content was observed under all treatments.

### Antioxidant enzyme activity

**Fig 7:**
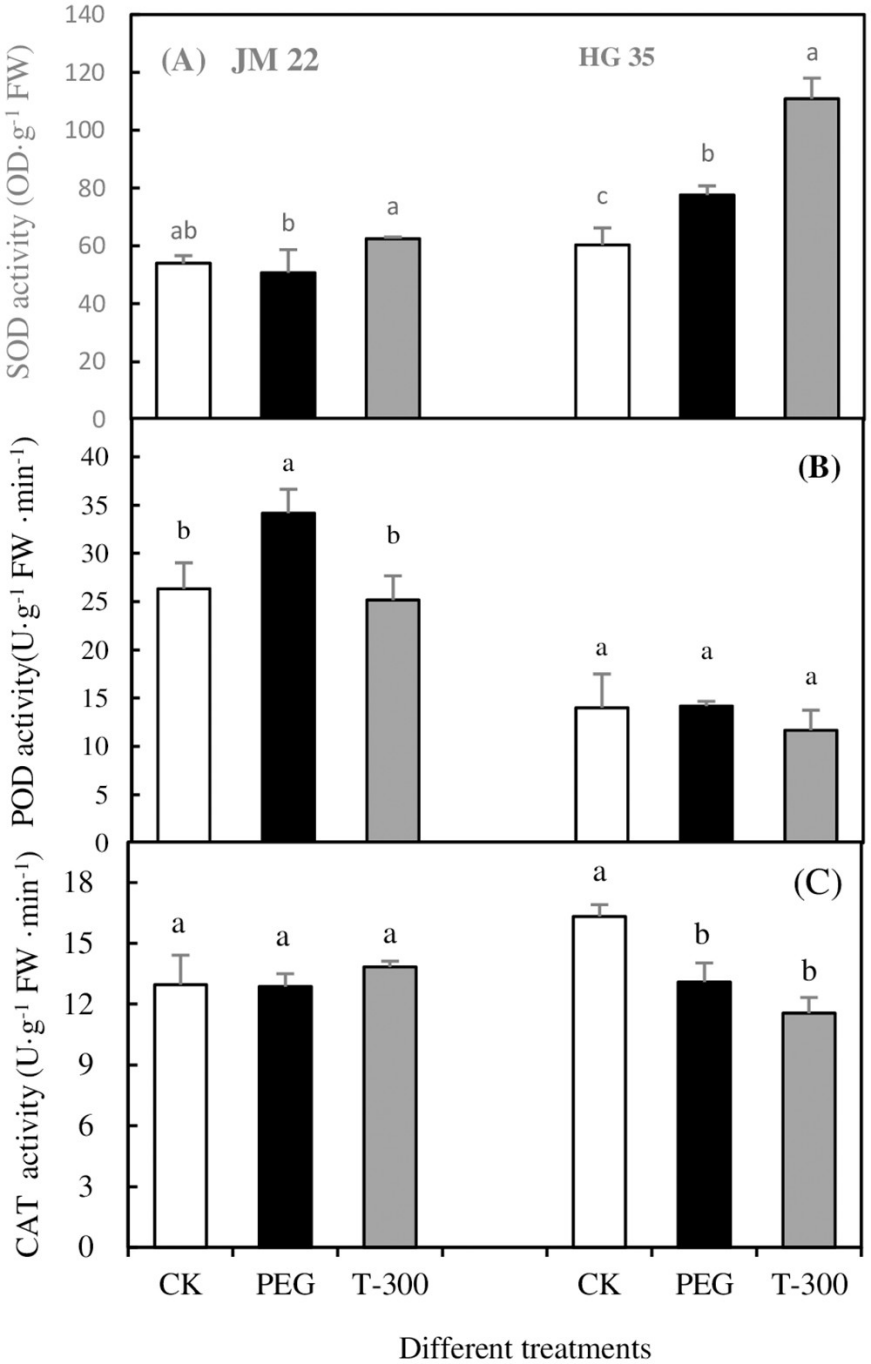
The effect of 300 μM melatonin on SOD (A), POD (B) and CAT (C) activity of two wheat varieties under different treatments. Different letters denote significant differences (p<0.05) within the same factor or interaction. Bars indicate mean relative expression values ± SE (n = 4).

Fig 7 shows different effects of melatonin on antioxidant enzyme activity of wheat leaf tissue under different treatments. The SOD activity of JM22 leaf tissue changed insignificantly, but SOD activity in HG35 leaf tissue increased significantly by 28.67% compared to the CK treatment. Compared to PEG alone, SOD activity increased significantly by 23.15% and 42.82%, respectively, in JM22 and HG35 (Fig 7A). POD activity of JM22 leaf tissue increased significantly under PEG comparing with CK. This value decreased to the same level as CK under the PEG plus 300 μM melatonin treatment. For HG35, POD activity was lower than JM22, and showed no significant differences among three treatments (Fig 7B). CAT activity in JM22 did not change significantly among the three treatments. For HG35, the CAT activity of leaf under PEG and PEG+300 μM melatonin treatments both decreased significantly, with rates decreasing by 19.86% and 29.25%, respectively comparing with the CK treatment. There were no obvious differences between PEG and PEG+300 μM melatonin treatments (Fig 7C).

## Discussion

Drought stress inhibits seed germination and plant growth [32, 33]. Exogenous melatonin plays an important role in improving seed germinations under different abiotic stress conditions such as water deficit [34], high temperature [35], cold [13], and salt stress [36]. In our study, the same result was observed, and the PEG simulated drought stress significantly decreased germination rate; applying higher concentration melatonin (300 μM) alleviated the adverse effects of drought stress on wheat seed germination (Table 2). Cui et al. [37] found that melatonin significantly ameliorated seed germination and seedling growth under PEG stress by improving energy production and activating a metabolic cascade related to autophagy, protein degradation, and other processes. It is known that intracellular proteins are often degraded to free amino acid, which are important osmotic solutes [16]. Serine, asparagine, methionine and lysine are associated with yield gap-based drought tolerance [38]. Melatonin-induced amino acids can be increased to enhance the cellular osmotic potential [37]. In this study, principal component analysis revealed significant change in the amino acid content of drought stressed wheat seeds during germination (Fig 4). A sharp reduction of Glu and Pro content in germinated seed could be the source of nitrogen during quiescent dry seed resuming metabolic activity. Once subjected to PEG stress, Ser, Pro and Glu contents increased in JM22 seeds; however, amino acids including Glu, His, Ser, Gly, Thr, Pro, Ala, Val, Met, Ile, Leu content were increased in HG35 seed. This indicated that drought hampered energy transformation and the supply of amino acid during germination. Applying 1–10 μM melatonin furtherly improved amino acids except Lys and Tyr content in JM22 seeds under PEG, which reveals the possible reason of deteriorating germination under the 1–10 μM melatonin treatment compared with PEG alone (S1 Table and Table 2); inversely, all amino acid levels except Cys content in HG35 seeds decreased under PEG plus melatonin. This could be interpreted partially for different drought-resisting mechanisms of these two varieties under PEG with or without melatonin involved. Lysine is an important amino acid that can be transformed into proline, aminobutyric acid, and polyamines during drought resisting processes [39–41]. Hartmann et al. [42] reported a pathogen-inducible L-Lys catabolic pathway in plants that generated N-hydroxy pipecolic acid as a critical regulator of systemic acquired resistance to pathogen infection. Also, histone deacetylase 14, played a significant role in deacetylation of lysine on histone, and was involved in melatonin biosynthesis of *Arabidopsis* thaliana [43]. Therefore, any decrease in lysine content during germination may indicate its transformation into other substances to raise the level of stress tolerance in plants. In this study, the changing trend of Lys content in two varieties was decreased under PEG, indicating its transformation into other substances to raise the level of stress tolerance in plants. It was increased under PEG plus 300 μM melatonin, suggesting melatonin retarded lysine’s transformation. Additionally, we also observed Lys content was significantly and positively correlated with germination percentage, germination index, germination potential, radicle length, and plumule length. All these indicated that Lys has an important role in regulation of melatonin on seed germination and crop growth. Either melatonin improve drought resistant by regulating metabolites directly or indirectly, changing amino acids is nonnegligible. The detailed interaction mechanism and melatonin content changing would be needed to reveal by further researches.

The regulatory effect of melatonin on osmotic solutes related not only to varieties, but also to melatonin concentrations [34, 44, 45]. In this study, melatonin at low concentration (1 or 10 μM) had no effect or increased adverse effects of PEG-induced drought stress on wheat seed germination. However, we found that 300 μM melatonin can alleviate symptoms of drought stress. This was not consistent with a study showing that exogenous melatonin at 100 μM gave the greatest germination rate of cucumber [8]. The discrepancy in these results may be due to different plant species having different levels of sensitivity to melatonin, and the response to melatonin in herbaceous plants was more intense than in woody plants [46]. Wang et al. [47] showed that low concentration of melatonin (1, 10 and 100 μM) had no effect on cotyledon size, while 1000 μM melatonin treatment caused smaller leaf size in *Arabidopsis* by reducing cell division rate, cell size and cell number. For cherry and *Brassica juncea*, exogenous applications of melatonin at low concentration (0–1 μM) could stimulate root growth, while higher concentrations (5–10 μM) inhibited or had no significant effect on root growth [48, 49]. These results also suggested that there were many influential elements including different effective concentrations and inhibiting concentrations for different plants. Another study found that melatonin (100 μM) priming does not affect soybean germination under normal condition [50]. Under drought stress, low concentration (1 μM and 10 μM) inhibited leaf physiology by communication of abscisic acid and hydrogen peroxide [27]. Sarropoulou et al. [49] pointed out that high melatonin concentrations (5 and 10 μM) increased the levels of proline and carbohydrates that helps osmoregulation in cherry leaves, while low melatonin concentrations (0.05, 0.1 and 1 μM) significantly reduced the proline content in root, promoting the synthesis of glycine and succinyl-CoA that influence porphyrins biosynthesis. They showed that melatonin at different concentrations may have different effects on synthesis and transportation of regulatory substances according to different plant parts and water conditions.

Therefore, melatonin at 300 μM can be deemed an effective concentration on wheat seed germination to resist drought, and to study its regulation on leaf physiology of wheat seedling. A melatonin treatment could increase drought tolerance by eliminating excess ROS of seedling [51]. The present study showed that membrane lipid peroxidation was alleviated by adding 300 μM melatonin under drought condition (Fig 6). Meantime, soluble protein content increased and adjusted osmotic pressure; and SOD activity was increased in JM 22 leaves. This result was consistent with that of Li et al. [44], who noted that exogenous melatonin can alleviate damage from drought stress in *Brassica napus* L. (rapeseed) seedlings by enhancing solute accumulation and activities of catalase, ascorbate peroxidase, and peroxidase. The effect of melatonin primarily increased SOD activity, while having a small effect on adjusting osmotic potential regulated by soluble proteins for Hengguan35-a drought resistant variety. MDA content of HG35 did not change significantly under all treatments. This is maybe possible due to different cell turgor and water holding capacity for wheat varieties, which was also increased by melatonin [34]. Higher cell turgor and water holding capacity can help cell membranes avoid being damaged easily from drought, then melatonin would not take obvious effect. In contrast, melatonin would take efficient impact on plant function disturbed by external disadvantages. Taken together, drought resistance in plants is a complex process, which can be regulated by melatonin related to a series of responses including osmotic pressure, antioxidant reaction, related gene expression. Regulation was a function of different varieties, water conditions, and plant parts [27, 49, 52].

## Conclusion

In conclusion, our study revealed that melatonin at 300 μM concentration alleviated the adverse effects of drought stress on seed germination and increased leaf antioxidant ability in wheat seedling. And there were different changings amplitude for wheat genotypic difference in drought resistance. HG35 showed better performance at germination, radicle length, radicle number and more stable physiological balance than that of JM22 under drought stress. Applying melatonin could improve JM 22 germination and physiological status more apparently by regulating amino acids content and transformation, such as glutamate, proline, and lysine. Especially for lysine, its content was significantly and positively correlated with the seed gemination indexes, maybe play a significant role in regulating drought resistance in wheat seeds treated with melatonin. Therefore, appropriate wheat variety and optimal concentration of melatonin selection is crucial to improve seedling quality and later production in drought region.

## Supporting information

S1 Table The objective value corresponding to Fig 5. (DOCX)
