# Supplementary material for: Analysis of melatonin regulation of germination and antioxidant metabolism in different wheat cultivars under polyethylene glycol stress
Source: PLoS One. 2020 Aug 13;15(8):e0237536. doi: 10.1371/journal.pone.0237536 (PMC7425870; doi:10.1371/journal.pone.0237536)
Supplement: S1 Table — (DOCX) [file pone.0237536.s001.docx]

**Table S1 Effect of different concentrations of melatonin on amino acid content in winter wheat under PEG treatments (mg kg^-1^).**

| **Cultivar** | **Treatment** | **Asp** | **Glu** | **His** | **Ser** | **Arg** | **Gly** | **Thr** | **Pro** | **Ala** | **Val** | **Met** | **Cys** | **Ile** | **Leu** | **Phe** | **Lys** | **Tyr** |
| --- | --- | --- | --- | --- | --- | --- | --- | --- | --- | --- | --- | --- | --- | --- | --- | --- | --- | --- |
| JM22 | Seed | 6.74 | 40.87 | 2.48 | 6.37 | 6.51 | 5.92 | 3.36 | 14.91 | 4.80 | 5.64 | 1.24 | 1.74 | 4.10 | 8.25 | 4.94 | 1.54 | 0.52 |
|  | CK | 7.66 | 23.71 | 1.88 | 4.81 | 6.84 | 5.31 | 2.18 | 10.50 | 4.71 | 5.36 | 1.04 | 2.18 | 3.94 | 7.31 | 5.33 | 3.53 | 1.20 |
|  | PEG | 4.70 | 34.24 | 1.57 | 4.99 | 5.18 | 4.87 | 1.82 | 13.60 | 3.67 | 4.55 | 0.96 | 2.15 | 3.47 | 6.94 | 4.85 | 1.82 | 0.76 |
|  | 1μM | 6.92 | 40.02 | 2.53 | 6.53 | 6.92 | 6.42 | 3.41 | 15.60 | 5.01 | 5.73 | 1.37 | 2.63 | 4.18 | 8.42 | 5.09 | 1.22 | 0.44 |
|  | 10μM | 6.84 | 42.68 | 2.78 | 6.46 | 6.99 | 6.29 | 3.26 | 15.52 | 4.95 | 5.77 | 1.41 | 2.74 | 4.30 | 8.67 | 5.49 | 1.70 | 0.49 |
|  | 100μM | 6.08 | 32.01 | 2.14 | 5.81 | 6.30 | 5.67 | 2.84 | 12.32 | 3.87 | 4.60 | 1.09 | 2.98 | 3.70 | 7.36 | 4.52 | 1.28 | 0.44 |
|  | 300μM | 6.67 | 34.09 | 2.05 | 5.40 | 6.27 | 5.28 | 2.54 | 13.70 | 4.50 | 5.35 | 0.94 | 1.96 | 4.00 | 7.70 | 5.10 | 2.19 | 0.65 |
|  |  |  |  |  |  |  |  |  |  |  |  |  |  |  |  |  |  |  |
| HG35 | Seed | 6.00 | 37.05 | 1.95 | 5.66 | 6.35 | 5.94 | 2.71 | 14.09 | 4.23 | 5.01 | 1.26 | 2.52 | 3.89 | 7.76 | 5.29 | 2.70 | 0.71 |
|  | CK | 7.26 | 24.09 | 1.86 | 4.33 | 6.96 | 5.02 | 1.85 | 10.53 | 4.65 | 5.39 | 1.03 | 1.99 | 4.01 | 7.57 | 5.84 | 4.64 | 1.41 |
|  | PEG | 6.40 | 36.61 | 2.15 | 5.94 | 6.44 | 5.80 | 3.12 | 16.35 | 4.88 | 5.56 | 1.17 | 1.52 | 4.21 | 8.37 | 5.22 | 1.73 | 0.66 |
|  | 1μM | 5.55 | 32.03 | 1.80 | 4.91 | 5.67 | 4.73 | 2.34 | 13.97 | 3.96 | 4.65 | 0.96 | 1.60 | 3.52 | 6.89 | 4.51 | 1.57 | 0.59 |
|  | 10μM | 5.31 | 29.12 | 1.65 | 4.64 | 5.84 | 5.00 | 2.15 | 12.02 | 3.84 | 4.26 | 1.07 | 2.24 | 3.14 | 6.26 | 4.09 | 1.22 | 0.76 |
|  | 100μM | 6.40 | 34.17 | 2.07 | 5.42 | 6.45 | 5.08 | 2.77 | 14.62 | 4.63 | 5.20 | 1.11 | 1.51 | 3.99 | 7.86 | 5.19 | 1.95 | 0.51 |
|  | 300μM | 5.88 | 29.63 | 1.78 | 4.83 | 5.83 | 5.00 | 2.31 | 12.67 | 4.09 | 4.75 | 1.00 | 2.07 | 3.63 | 7.09 | 4.85 | 1.85 | 0.59 |

seed represent no-germinated seeds; CK represent seeds germinated by distilled water; PEG represent seeds germinated by 20%PEG; 1μM, 10μM, 100μM, and 300μM, represent seeds germinated by 1μM, 10μM, 100μM, and 300μM melatonin plus 20% PEG, respectively
